# Early Outcomes of Hirschsprung's Disease after Definitive Surgery: A Ten-year Experience

**DOI:** 10.2174/0115733963271997240605103909

**Published:** 2024-06-20

**Authors:** Muntadhar Muhammad Isa, Maimun Syukri, Muchlisin Zainal Abidin, Dian Adi Syahputra, Teuku Yusriadi, Yumna Muzakkir, Siti Magfirah, Gunadi Gunadi

**Affiliations:** 1 Graduate School of Mathematics and Applied Sciences, Syiah Kuala University, Banda Aceh, Indonesia;; 2 Pediatric Surgery Division, Department of Surgery, Syiah Kuala University/Dr. Zainoel Abidin General Hospital, Banda Aceh, Indonesia;; 3 Division of Nephrology and Hypertension, Department of Internal Medicine Faculty of Medicine, Syiah Kuala University/Dr. Zainoel Abidin General Hospital, Banda Aceh, Indonesia;; 4 Faculty of Marine and Fisheries, Syiah Kuala University;; 5 Medical Faculty, Syiah Kuala University/Dr. Zainoel Abidin General Hospital, Banda Aceh, Indonesia;; 6 Pediatric Surgery Division, Department of Surgery, Gajah Mada University/Dr. Sardjito Hospital, Yogyakarta, Indonesia

**Keywords:** Hirschsprung’s disease, early outcome, definitive surgery, transanal endorectal pull-through, complication, developing country

## Abstract

**Introduction:**

This study aimed to examine the characteristics of Hirschsprung’s Disease (HD) in patients aged<18 who underwent surgical procedure at Dr. Zainoel Abidin (RSUDZA) General Hospital, Banda Aceh, Indonesia, between January 2010 and December 2020.

**Methods:**

This retrospective study collected and analyzed data from medical records of 18-year-old or younger children (n = 180) diagnosed with HD at RSUDZA. The surgical procedures included the Duhamel procedure, Soave procedure, the Soave Transanal Endorectal Pull-through (TEPT) procedure, and the Swenson TEPT procedure. Early outcomes of the surgery were then compared between males and females. The comparrative analysis was determined based on Chi-square analysis, where *p*<0.05 was considered significant.

**Results:**

There were 111 (61.7%) male patients and 69 (38.3%) female patients, with a mean age of 15.2 months. The Soave TEPT is the most frequently performed procedure (91.7%). Emerging clinical manifestations include constipation (176; 97.8%) and soiling (171; 95%). Preoperative barium enema and postoperative pathological examination confirmed that almost all patients (99.4%) had an aganglionic segment confined to the rectosigmoid area. The average length of operation was 69.7 ± 65 minutes and average bleeding time was 5.4 ± 34 mL. The average discharge time was 3.3 ± 73.3 days. No significant difference was found in post-surgery complications between males and females (*p*>0.5). The immediate complications were not associated with surgical methods (*p* = 0.83).

**Conclusion:**

Our descriptive study has suggested the Soave TEPT technique as appropriate to manage HD.

## 1 INTRODUCTION

Hirschsprung’s Disease (HD) is a congenital abnormality characterized by the absence of ganglion cells in the submucosa (Meissner) and myenteric (Auerbach) plexuses of the terminal rectum [1-3]. HD is common among children, affecting 1 in every 5000 births, with a boy-girl ratio of 4:1-3:1 [4-6]. The Enteric Nervous System (ENS) controls colon peristalsis under normal movement conditions [7-9]. ENS regulates Gastrointestinal (GI) tract physiology *via* network nerves located inside the colon wall and the myenteric and submucosal ganglia [10, 11]. The absence of ganglion cells causes persistent spastic contraction, which can manifest clinically as functional colonic obstruction [12]. This occurs due to an error in ENS development during fetal life, which disrupts the migration process of cell cristae (ectoderm) of the side neural tube to the colon wall, particularly through the vagal trunk. The process is ongoing in fetuses aged 5 to 12 weeks [13-17].

The emergence of clinical manifestations varies according to the location of the aganglionic segment. Lateness expenditure meconium (>48 hours), abdominal distension, and biliary emesis are common symptoms in neonates [2, 3, 14]. Patients with clinical manifestations of HD must undergo a diagnostic evaluation method. The HD diagnostic workup includes three tests. First, the presence of a transitional zone is the critical feature that indicates HD in a Contrast Enema (CE) test. The rectoanal inhibition reflex is evaluated by Anorectal Manometry (ARM) [18-20]. Failure to elicit this reflex indicates HD. In the case of HD, the third option is a Rectal Suction Biopsy (RSB), which reveals elevated Acetylcholinesterase (AChE) activity and aganglionosis. The gold standard, however, is a rectum Full-thickness Biopsy (FTB), which provides the most definitive answer. An FTB with no ganglion cells confirms the diagnosis of HD and the need for treatment, including definitive surgery [21-24]. The principle entails anastomoses of the colonic ganglion near the sphincter ani to allow for feces expenditure. Most patients can achieve adequate colon function after surgery, despite the residual colon ganglion being slightly abnormal [25, 26]. Some standard procedures for HD have been reported to be performed, including transabdominal endorectal pull-through (Soave), Duhamel, Transanal Endorectal Pull-through (TEPT), Swenson TEPT, and posterior neurectomy procedures with varying results [4, 27-33]. Thus, this study was conducted to review the early outcomes of HD patients undergoing definitive procedure surgery at Dr. Zainoel Abidin General Hospital (RSUDZA), Indonesia.

## 2 METHODS

### 2.1 Study Design

This study involved the descriptive observational design with data collected retrospectively. Data were obtained from medical records of patients hospitalized at RSUDZA, Banda Aceh, Indonesia, from January 2010 to December 2020 (ten years).

### 2.2 Study Subjects

Subjects were recruited *via* total sampling, where all eligible patients (n = 180) were treated as samples of this present study. Patients aged 18 years old or below and who have undergone definitive surgical procedures, such as Duhamel, Soave, TEPT (Soave), and TEPT (Swenson) were included. The definition of Hirschsprung's disease was based on the International Classification Disease-10 code (ICD-10) (Q43.1). Clinical features, barium enema, and histopathological examination confirmed the diagnosis of HD in all patients.

### 2.3 Data Collection

The data were collected from medical records, namely age, gender, clinical manifestations, histological examination findings, preoperative preparation, operative details, and immediate post-operative complications. For age parameters, the patients were grouped into neonates (<1 month), infants (1 month-1 year), toddlers (1-3 years), children (3-10 years), and adolescents (10-18 years). Recorded clinical manifestations included abdominal distension, bile vomiting, delay in meconium elimination > 24 hours, constipation, fever, stool squirting, and soiling. HD cases were divided into three types according to the aganglionosis, namely short-segment, long-segment, and total colon aganglionosis. Time required to complete the opeartion and volume of blood loss during the operation were noted. The number of days between the day of patient admission and hospital discharge was also acquired from the medical records.

### 2.4 Statistical Analysis

Statistical software (SPSS version 26.0) was employed to analyze the data. Continuous data have been expressed as mean ± standard deviation (SD), while categorical data have been expressed as frequency. Data were divided between males and females to assess the effect of gender on the early outcomes. The early outcomes were also divided based on operative techniques [Duhamel, Soave, TEPT (Soave), and TEPT (Swenson)]. The statistical difference was determined by the Chi-square test, with a *p*-value <0.05.

## 3 THE OPERATIVE TECHNIQUE

The operative techniques for managing HD in RSUDZA, as reported in the present study, are uncommon among Indonesian hospitals. The techniques involve a one-stage Soave TEPT procedure. These techniques can reduce treatment costs and shorten the patient's treatment period. Prior to the pull-through, in this study, all patients underwent a rectal biopsy to confirm the diagnosis, and all patients were given a contrast enema to determine the location of the transition zone. During the preoperative period, bowel preparation was not conducted for the surgery. Instead, only a routine washout with NaCl 0.9% at a dosage of 20 cc per kilogram of body weight, along with a preoperative washout, was administered. The washout administered on the operating table adequately cleansed the intestines of all patients.

**Fig. (1):**
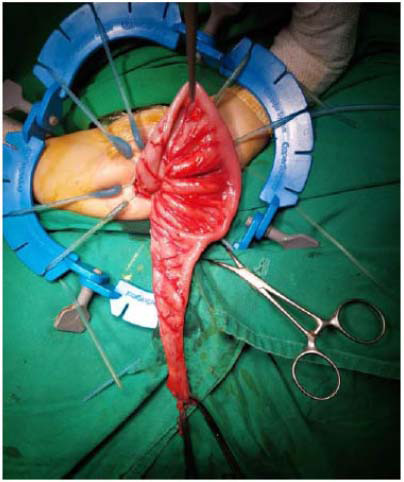
Visualizing the transanal endorectal pull-through technique. Source: Documentary of Pediatric Surgery Division, Department of Surgery, Syiah Kuala University/Dr. Zainoel Abidin General Hospital.

The procedure was entirely transanally performed (TEPT). Following general anesthesia and the placement of intravenous lines, the patients were given a third-generation cephalosporin intravenously. Rectal irrigation was performed with a dilute NaCl solution. A lithotomy was then performed on the patient. On a regular basis, a bladder catheter was inserted. To expose the anal canal, the Lone Star anal retractor was used. Submucosal epinephrine or saline injections were rare. A stitch suture was made at 1 cm proximal to the dentate line, and then a circular incision made 0.7 cm proximal to the line penetrated the mucosa. The depth was ascertained by the penetration of the submucosal layer. Thereafter, the mucosectomy was performed until reaching the peritoneal cuff reflexion. A circumferential row of 4-0 silk stay sutures was placed about 0.5 to 1 cm above the dentate line (Fig. **1**) [16, 34, 35].

The rectal mucosa was incised just distal to the traction sutures and lifted circumferentially with a fine diathermy needle to develop the submucosal plane. After establishing the submucosal plane, the dissection was easily continued proximally with blunt dissection and cauterization of submucosal infiltrating vessels. The traction on the mucosal tube aided in the proximal extension of the mucosal dissection until it reached the level proximal to the peritoneal reflection (approximately 10 to 15 cm above the dentate line). Four stay sutures were used to control the upper end of the muscular cuff, which was circumferentially incised to allow access to the full-thickness sigmoid colon.

The colonic dissection was performed by utilizing a harmonic scalpel. The dissection proceeded toward the histologically confirmed transition zone, with rectosigmoid vessels cauterized and divided accordingly. The long seromuscular cuff was inverted outside the anus and shortened to less than 5 cm in length before being returned to its normal position. Following resection of the aganglionic segment, the normally innervated bowel was pulled through the muscular cuff and anastomosed to the remaining mucosa above the dentate line with 4-0 slowly absorbable suture material. Feeding was permitted 3 hours after the surgery. If no complications arose, the patients were discharged 3-5 days after the surgery. The first rectal digital examination was performed 14 days later, and Busi Hegar anal dilator was employed twice daily every 14 days, 28 days, and 42 days post-surgery for routine anal dilation.

## 4 RESULTS

The characteristics of the subjects included in the present study are provided in Table **1**. Of 180 HD patients in this study, 111 (61.7%) were male and 69 (38.3%) were female. The study population comprised with an average age of 15.2 months (<1 month; n = 53, 29.4%), infants (1 month-1 year; n = 68, 37.8%), toddlers (1-3 years; n = 11, 6.1%), children (3-10 years; n = 11, 6.1%), and adolescents (10-18 years; n = 2, 1.1%). The most common type of aganglionosis was short-segment aganglionosis in 179 (99.4%) cases, followed by long-segment aganglionosis (n = 1, 0.6%). Clinical manifestations, particularly constipation, occurred in 176 (97.8%) patients, soiling in 171 (95%) patients, and abdominal distension in 168 (93.3%) patients. A total of 165 (91.7%) patients underwent the TEPT (like soave) procedure, with an average operation time of 69.7 ± 65 minutes and average blood loss of 5.4 ± 34 mL. The average length of stay was 3.3 ± 73.3 days.

One hundred and eighty patients underwent definitive surgery. Histopathology confirmed HD in all cases. We found 61.1% of male patients with short segment HD and 38.30% of females (Table **2**). The TEPT (Soave) procedure was the most frequently performed operation (91.7%), followed by Soave (4.4%), Duhamel (2.8%), and TEPT procedures (Swenson) (1.1%). Almost all patients had perianal excoriation, partial stenosis, and post-surgery soiling as complications after the surgery (Table **2**). Among 165 patients who underwent TEPT, 156 (94.5%) had post-surgery soiling and 163 (98.8%) had partial stenosis. According to statistical analysis, there were no significant differences in immediate complications between males and females (*p*>0.5) (Table **2**). Among the performed surgical procedures, the complication occurrences were similar, including soiling (*p* = 0.83) and partial stenosis (*p* = 0.98) (Table **3**).

## 5 DISCUSSION

Demographic data of our patients in this present report have suggested a higher prevalence of HD among male patients and those aged between 1 month to 1 year. In line with previous studies, male patients were predominant [36]. The male-to-female ratio was 71:15 in a similar study reported from India [37]. In a study from the United States of America (USA) that specifically included patients with late diagnosis of HD, the male-to-female ratio was 19:9 [36]. In a larger study from the USA, the number of male patients was over three times higher than female patients (974 vs. 306) [38]. In terms of age, patients in our present study were younger than those reported in some previous studies [36]. However, in another study comprised of 55 cases, the number of patients aged < 1 months was higher as compared to other age groups. In the present study, we also found constipation to be the most common clinical manifestation (97.8%), followed by soiling (95%) and abdominal distension (93.3%). Such manifestations are similar to those previously reported [36]. The presentation of short-segment aganglionosis was prevalent in this present study, similar to that previously reported [39, 40].

Herein, the histological examination was employed as the gold standard for diagnosing HD. Because there are usually fewer ganglion cells 0.5-1.0 cm above the dentate line, the rectal biopsy should be taken at least 1.0-1.5 cm above it. A biopsy taken in close proximity to the aganglionic segment may inadvertently miss detecting a short aganglionic segment [41, 42]. In addition to hematoxylin and eosin, many pathologists conduct staining for acetylcholinesterase, which exhibits a distinct pattern in the submucosa and mucosa of children with HD. However, in cases reported in this present study, only immunochemical staining with hematoxylin and eosin was performed [43-46]. For an experienced pathologist, conventional hematoxylin and eosin-stained sections are frequently sufficient to rule out HD or confirm the diagnosis [6, 46-50]. In the present study, all patients were hystologically confirmed for positive HD. Surgical pathology reports for suction rectal biopsies involve adequate examination of the biopsy tissue and explicit statements regarding the presence or absence of identifiable submucosal ganglion cells and sub-mucosal nerve hypertrophy [51-53].

For the surgical treatment of HD, several surgical techniques are used to resect the distal aganglionic colon. The surgical management now has moved from multi-stage open procedures to single-stage transanal techniques [43, 54-56], and the TEPT procedure has become one of the most frequently performed surgery in RSUDZA. Apart from TEPT, which was commonly used in this study, a few patients underwent surgery with either the Duhamel or Soave procedures. The choice of surgery was not dependent on the seriousness of the case, but rather on the surgeon's skills and experience with the specific techniques. The TEPT procedure, first developed by De La Torre and Langer, was a Soave- like transanal submucosal dissection with an endorectal pull through, leaving an aganglionic rectal muscular cuff [54, 57, 58]. The TEPT procedure was associated with a shorter length of post-operation hospital stay [32, 59].

The most common measures of outcomes for HD following the surgical procedure are continence and constipation [60-62]. Herein, among all patients who underwent TEPT, 156 (94.5%) had post surgical excoriation, and 163 (98.8%) had partial stenosis [63-66]. The Soave TEPT technique commonly results in the formation of the residual cuff, contributing to partial stenosis. This complication often responds well to treatment with anal dilatation, a recognized therapeutic approach in clinical practice [67-69]. In cases reported herein, constipation was also found, but it resolved spontaneously. Soiling and constipation due to partial stenosis were the most common complications after the patient underwent TEPT.

It is worth noting that in trans anal approach, the sphincter needs to be stretched [70-73]. Anastomosis was made above the dentate line, but still, there was some injury to mucosa below the dentate line. The injury might occur due to the lack of surgical precision or because of the patient conditions of having tissue tension or inflammation, which contribute to injury susceptibility. The consequence of having a mucosal injury below the dentate line is the increased risk of incontinence, accompanied by unfavorable outcomes [74-76]. Efforts should be made to avoid mucosal injury extending beyond the distal dentate line during the surgery. In case of such an injury, prompt repair is imperative. These factors may adversely affect continence. Previously, cases of constipation were reported after the surgery in HD, and sometimes, multidisciplinary approach following the surgery was required [41, 42].

Our study has provided descriptive data on early surgical outcomes in HD management, where the Soave TEPT is the most frequent technique used. Further studies are warranted to evaluate the surgical options for treating this disease. Based on our experience, long-term observation post- surgery is required for any functional outcomes. Additionally, an investigation of the multidisciplinary approach to improve the recovery process is recommended [48, 77].

## CONCLUSION

The outcomes of HD patients that predominantly underwent Soave TEPT (90% of the total cases), included perianal excoriation, partial stenosis, and post-surgery soiling. More than 90% of the total patients receiving surgical management with the Soave TEPT technique experienced partial stenosis, post-surgery soiling, or both. This report suggests the Soave TEPT to be a relatively safe procedure considering that no serious complications or post-operative deaths were found.

## AUTHORS’ CONTRIBUTIONS

The authors confirm their contribution to the paper as follows: study conception and design: M.M. Isa, D. A. Syahputra and G. Gunadi; data collection: Y. Muzakkir; analysis and interpretation of results: M. Syukri, M. Zainal Abidin and S. Magfirah; draft manuscript: T. Yusriadi. All authors reviewed the results and approved the final version of the manuscript.

## Figures and Tables

**Table 1:** Clinical characteristics of patients with HD.

**Characteristics**	**n (%)**
Gender	-
Male	111 (61.7)
Female	69 (38.3)
Age	-
Neonate (<1 month)	53 (29.4)
Infant (1 month-1 year)	68 (37.8)
Toddler (1-3 years)	45 (25)
Child (3-10 years)	11 (6.1)
Adolescent (10-18 years)	3 (1.7)
Clinical manifestations	-
Abdominal distension	168 (93.3)
Bilious vomiting	71 (39.4)
Delayed passage of meconium	155 (86.1)
Constipation	176 (97.8)
Fever	4 (2.2)
Stool squirting	160 (88.9)
Soiling	171 (95)
Operation method	-
Duhamel procedure	5 (2.8)
Soave procedure	8 (4.4)
TEPT (Soave)	165 (91.7)
TEPT (Swenson)	2 (1.1)
Histopathology	-
Aganglionosis	180 (100)
Type of aganglionosis	-
Short segment	179 (99.4)
Long segment	1 (0.6)
Total colon aganglionosis	0
Operative time (min), mean ± SD	69.7 ± 65
Operative blood loss (mL), mean ± SD	5.4 ± 34
Average hospital stay (days), mean ± SD	3.3 ± 73.3

**Table 2:** Characteristics of HD patients.

**Characteristics**	**n (%)**			**Chi-square *p-value***
	**Total (n = 180)**	**Male (n = 111)**	**Female (n = 69)**	
**Age**	-	-	-	0.514
**Neonate (<1 month)**	53 (31.2)	33 (18)	20 (11)	-
**Infant (1 month-1 year)**	68 (37.8)	36 (20)	32 (18)	-
**Toddler (1-3 years)**	45 (25)	33 (18)	12 (6.7)	-
**Child (3-10 years)**	11 (6.1)	7(3.9)	4 (2.2)	-
**Adolescent (10-18 years)**	3 (1.7)	2 (1.1)	1 (0.6)	-
**Type of HD**	-	-	-	-
**Short segment**	179 (99.4)	110 (61.1)	69 (38.3)	0.429
**Long segment**	1 (0.6)	1 (0.56)	0 (0.0)	-
**Surgical procedure**	-	-	-	-
**Duhamel procedure**	5 (2.8)	3 (1.7)	2 (1.1)	0.528
**Soave procedure**	8 (4.4)	3 (1.7)	5 (2.8)	-
**TEPT (Soave)**	165 (91.7)	103 (57.2)	62 (34.4)	-
**TEPT (Swenson)**	2 (1.1)	2 (1.1)	0 (0.0)	-
**Early outcome after surgery**	-	-	-	-
**Perianal excoriation**	180 (100)	111 (61.7)	69 (38.3)	0.52
**Partial stenosis**	178 (98.9)	109 (60.6)	69 (38.3)	0.73
**Soiling**	171 (95)	106 (58.9)	65 (36.1)	0.59

**Table 3:** Early outcome of HD after definitive surgery.

**Definitive Surgery and Early Outcome after the Surgery**	**Yes**	**No**	***p-value***
**Soiling**	-	-	-
**Duhamel procedure**	5	0	0.83
**Soave procedure**	8	0	-
**TEPT (Soave)**	163	2	
**TEPT (Swenson)**	2	0	
**Partial stenosis**	-	-	0.98
**Duhamel procedure**	5	0	-
**Soave procedure**	8	0	
**TEPT (Soave)**	156	9	
**TEPT (Swenson)**	2	0	

**Abbreviation:** TEPT, Transanal endorectal pull-through.

## Data Availability

The datasets created or analyzed in this study will be provided by the corresponding author upon a reasonable request.

## LIST OF ABBREVIATIONS

ENS: Enteric Nervous System
GI: Gastrointestinal
HD: Hirschsprung’s Disease
TEPT: Transanal Endorectal Pull-through

## STANDARDS OF REPORTING

STROBE guidelines were followed.
